# Comparative Evaluation of Recombinant Protein Production in Different Biofactories: The Green Perspective

**DOI:** 10.1155/2014/136419

**Published:** 2014-03-12

**Authors:** Matilde Merlin, Elisa Gecchele, Stefano Capaldi, Mario Pezzotti, Linda Avesani

**Affiliations:** Department of Biotechnology, University of Verona, 37134 Verona, Italy

## Abstract

In recent years, the production of recombinant pharmaceutical proteins in heterologous systems has increased significantly. Most applications involve complex proteins and glycoproteins that are difficult to produce, thus promoting the development and improvement of a wide range of production platforms. No individual system is optimal for the production of all recombinant proteins, so the diversity of platforms based on plants offers a significant advantage. Here, we discuss the production of four recombinant pharmaceutical proteins using different platforms, highlighting from these examples the unique advantages of plant-based systems over traditional fermenter-based expression platforms.

## 1. Introduction

The market for recombinant pharmaceutical proteins is expanding rapidly. Indeed, nearly all pharmaceutical companies with a market capitalization value of more than $US 10 billion report that their revenue share from such products is growing faster than the share from small-molecule drugs [1]. The industry has focused on a small number of production platforms based on the bacterium* Escherichia coli*, several species of yeast, and a selection of insect and mammalian cell lines, which have been developed and improved in line with current good manufacturing practice (cGMP). However, focusing on a small number of platforms means that the unique requirements of certain target proteins are difficult to meet; this is the case of recombinant proteins that are required in small quantities (e.g., for individual patients) or in massive quantities or that need rapid production scale-up. Plant biotechnology can overcome some of these limitations and the potential of plant-based platforms for the flexible, low-cost production of high-quality, bioactive recombinant proteins is well-documented [2].

Plants successfully perform the majority of posttranslational modifications required for the activity of complex eukaryotic proteins and provide tremendous flexibility in terms of scale, cost, safety, and regulatory issues. For example, cell-based bioreactor systems including plant suspension cells and algae are ideal for lower-volume products, whereas field-grown commodity crops can produce metric tons of recombinant protein at highly competitive costs. Contained production systems based on plants have biosafety advantages over microbial and mammalian production platforms because they neither do produce endotoxins nor do they support the growth of pathogens that infect animals, thus reducing purification costs and minimizing the likelihood of facility shutdowns, decontamination issues, and supply limitations that lead to unmet patient/customer demands. Although the costs of downstream processing and purification are comparable in microbial, mammalian, and plant-based platforms, the lower up-front investment required for commercial production in plants and the potential economy of scale provided by cultivation over large areas are key advantages.

This combination of low capital investment, low-cost of goods, and highly scalable manufacturing means that many proteins that are unsuitable for production in fermenters can be produced commercially using plants. Other proteins can be produced more efficiently by fermentation in plant cells because the posttranslational modifications can be engineered to improve product quality and activity. Not all pharmaceuticals will benefit from plant-based systems but the best production platform should be determined empirically for each protein using a case-by-case approach. Several recent reviews have discussed the merits of plant-made pharmaceuticals [3], including specific issues related to commercial production [4] and considerations of cGMP issues in plants [5]. This review will focus on four target molecules that highlight different applications across a range of expression systems to illustrate important ways in which plant-based expression platforms are evolving to meet a spectrum of research, development, and commercial needs.

## 2. Human Glutamic Acid Decarboxylase 

The 65-kDa isoform of human glutamic acid decarboxylase (hGAD65) is an enzyme containing the prosthetic group pyridoxal 5′-phosphate (PLP). It forms obligate functional dimers and is localized in pancreatic *β*-islet cells as well as the brain, where it catalyzes the conversion of glutamate to *γ*-aminobutyric acid (GABA) and carbon dioxide. In human cells, the major pool of hGAD65 exists as an autoinactivated apoenzyme [6]. The crystal structure provides insight into both the molecular mechanism of catalytic activity and the structural determinants of its antigenicity [6].

The hGAD65 protein functions as an autoantigen in several autoimmune diseases, including autoimmune type 1 diabetes (T1D) and Stiff-Person syndrome. T1D is strongly associated with autoreactivity to hGAD65. Indeed, hGAD65 autoantibodies are present before the clinical onset of the disease and provide a useful marker to predict the likelihood of its development [7]. The relevance of such markers has been confirmed unequivocally in many laboratories that participate in the Diabetes Autoantibody Standardization Program (DASP), which is a collaboration between the US Centres for Disease Control and Prevention and the Immunology of Diabetes Society [8].

The autoantibodies are not directly pathogenic, whereas T cells play a dominant role in the initiation and progression of T1D. T-cell responses against the linear epitopes of hGAD65 can be detected in animal models of the disease and in humans at risk of T1D. Studies in animal models have shown that exposure to hGAD65 may induce immunotolerance [9, 10]. A phase II human clinical investigation, involving genetically predisposed children and young adults with multiple islet cell autoantibodies, is currently exploring whether treatment comprising two injections of 20 *μ*g doses of alum-formulated hGAD65 (the GAD vaccine, Diamyd Medical) prevents the onset of the disease (NCT01122446).

The prevalence of T1D in the general population is currently 0.04%, but this is increasing at 3% per annum in children. If the clinical trial discussed above is successful, then the global demand for recombinant hGAD65 would increase dramatically. GAD65 was initially sourced from porcine brains, although the most abundant source is monkey brain, with a yield of 12 mg/g [11]. These sources are not suitable for therapeutic GAD65 due to the risk of infection with prions and other pathogens, so heterologous production techniques were investigated following the isolation of hGAD65 cDNA [12].

In all heterologous systems, the yield of hGAD65 is reported by measuring its enzymatic and immunochemical activity. Posttranslational modifications occur in the N-terminal region, that is, blockage of the N-terminal amino group, palmitoylation, and phosphorylation, but none of these modifications are necessary for catalytic activity or immunogenicity so in theory the protein can be produced using any expression platform [11]. Furthermore, the abolition of enzymatic activity to generate a mutant protein (hGAD65mut) does not affect the immunoreactivity of the protein and thus its diagnostic and therapeutic potential [13].

Current commercial platforms for the production of diagnostic and research-grade GAD65 include yeast, baculovirus-infected insect cells, and wheat germ lysates, with costs of €2,000–60,000/mg. The suitability of these different production platforms has been discussed. For example, the expression of GAD65 in bacteria produced a misfolded protein that was primarily localized in inclusion bodies, and it was only possible to produce a soluble and immunogenic product by expressing the protein as an N-terminal fusion with thioredoxin or glutathione S-transferase [14, 15]. As well as making the protein soluble, the fusion partners also facilitated protein isolation, resulting in yields of up to 12.5 g/L.

Recombinant hGAD65 has also been expressed in baby hamster kidney (BHK) cells (Heinaes et al., unpublished data), Chinese hamster ovary (CHO) cells, and mouse myeloma cells, the latter resulting in the highest yield of 1.7 mg/L [16]. Although the overall yield was lower than achieved in bacteria, the recombinant protein was soluble and retained its native structure without a fusion partner. CHO cells have therefore been used to study the subcellular trafficking and localization of hGAD65.

Recombinant hGAD65 has also been expressed in the yeasts* Saccharomyces cerevisiae* and* Pichia pastoris*, both of which produced an active protein with yields of up to 3.52 mg/L [17]. Insect cells infected with baculovirus vectors achieved the highest yields of hGAD65 ever reported, in the best cases reaching 50 mg/L [18], but when hGAD65 was expressed with a C-terminal His_6_ tag, the yield dropped to 3–5 mg/L [19].

Several plant-based platforms have also been used to produce hGAD65.* Chlamydomonas reinhardtii* chloroplasts were transformed with an hGAD65 vector and the immunoreactive recombinant protein accounted for 0.3% of the total soluble protein (TSP) in the algal cells [20]. Immunoreactive and enzymatically active hGAD65 has also been expressed in tobacco and carrot plants albeit with disappointing yields; for example, in T1 tobacco plants, the yield was 10.5 *μ*g/g fresh weight (FW) in the leaves [21–23].

The production of hGAD65 in plant- and insect cell-platforms was achieved by expressing the catalytically inactive version, hGAD65mut, which retains its immunogenicity. The mutant protein accumulates to higher levels than its active counterpart, that is, up to 143.6 *μ*g/g FW in tobacco leaves [23]. The hGAD65mut mutant was generated by substituting the lysine residue that binds the cofactor PLP with an arginine residue (K396R). It was proposed that the wild-type version of hGAD65 interferes with plant cell metabolism to suppress its own synthesis, whereas the catalytically inactive version escapes such feedback and accumulates to higher levels.

Other modified versions of GAD65 have been expressed, including a soluble form generated by substituting the N-terminal domain with the homologous region of the soluble 67-kDa isoform of the protein. This substitution increased the yields of the protein from 3.52 to 12.16 mg/L in* S. cerevisiae* [17] and from 10.5 to 50 *μ*g/g in tobacco leaves [24]. Although differences in the stability of the N-terminal *α*-helical regions could theoretically account for these differences, there was no improvement to the yield of hGAD65mut in plants when the modification was included [25]. This suggests that abolishing the membrane interactions by removing the N-terminal region does not cause any additional benefit when the biological activity of the protein is eliminated.

Modifying the protein for retention in the endoplasmic reticulum (ER) of plant cells did not increase its yield in transgenic tobacco plants [25]. GAD67/65mut was also expressed in the seeds of three different species (Arabidopsis, tobacco, and petunia) and retained in the ER. The highest yield of 4.5 mg/g dry weight (DW) was achieved in Arabidopsis seeds [26].

The purification of hGAD65 from yeast, insect, and mammalian cells is usually achieved by immunoaffinity chromatography using anti-GAD monoclonal antibodies [16, 27], anion- exchange chromatography [18], or a combination of the two [17]. In bacteria and yeast, higher yields were achieved by expressing tagged fusion proteins and using the tag as the affinity ligand [15, 19, 28]. Although the purification of hGAD65 has not been reported in plants, edible plant tissues containing the protein can be administered by oral delivery such that extensive purification is not required. It has been demonstrated that the oral administration of a crude transgenic tobacco extract containing hGAD65, in combination with interleukin-4 (IL-4), diminished the peripheral immune response to a subsequent systemic challenge with the same autoantigen by inducing oral tolerance [21].

## 3. Norwalk Virus-Like Particles

Norwalk virus (NV) is the prototype human norovirus (NoV), which contains a single-stranded, positive-sense nonenveloped RNA genome containing three open reading frames and a polyadenylate tail [29]. The NV capsid is a 38 nm icosahedral structure assembled from 90 dimers of VP1, the 58-kDa capsid protein (CP), with *T* = 3 symmetry [30, 31]. NoV belongs to a group of highly infectious viruses that are responsible for more than 95% of epidemic outbreaks of viral gastroenteritis in adults in developed and developing countries [32]. In the USA alone, NoV causes ~21 million infections per year, resulting in 70,000 hospitalizations and 800 deaths, at a cost of $US 5.5 billion [33] (https://www.bcm.edu/molvir). In developing countries, NoV is responsible for up to 1.1 million hospitalizations annually and 218,000 deaths among children [32].

The increasing recognition of NoV as a disease agent, the absence of a specific treatment, and the limited success in preventing disease outbreaks have led to the evaluation of virus-based vaccines [34]. However, the insufficient quantity of virus particles available for analysis has delayed the development of such a vaccine. The only natural source of NV particles is human stools, which characteristically contain very low concentrations of viruses [29].

The successful cloning, sequencing, and expression of the major NV capsid protein VP1 in insect cells were a major breakthrough and showed that recombinant VP1 folds spontaneously into empty Norwalk virus-like particles (NVLPs) that are stable following lyophilization at temperatures of up to 55°C and/or when exposed to acids (pH 3–7) [35]. The recombinant NVLPs remain immunogenic and interact with cellular receptors, eliciting a strong host immune response against the virus [29, 31, 36, 37], and would therefore make ideal NV vaccine candidates [38, 39].

Preclinical studies showed that recombinant NVLPs are immunogenic when administered by the parenteral [29], oral [40, 41], and intranasal routes [42]. Furthermore, a specific formulation for intranasal delivery, comprising NVLP dry powder and a novel plant-derived polysaccharide with gelling properties (GelSite), showed superior immunogenicity in mice than in a liquid formulation including an adjuvant [43].

In phase I studies, orally administered NVLPs were found to be safe but only modestly immunogenic as determined by measuring serum antibody levels and counting specific antibody-secreting cells (ASCs) [44–46]. Conversely, a nasally delivered NVLP formulation including an adjuvant was well tolerated and highly immunogenic [47]. A phase I/II study carried out by LigoCyte Pharmaceuticals showed that two 50 *μ*g intranasal doses of NVLPs protected mice against challenge with a homologous virus [48]. Furthermore, parenteral administration in phase I/II studies demonstrated that two 100 *μ*g intramuscular doses of NVLP vaccine were well tolerated and produced a clinically relevant impact on the incidence of NV after challenge, as well as the severity in breakthrough cases (Takeda Pharmaceuticals USA Inc., 2013). Collectively, these clinical trials indicated that vaccination may be useful to prevent disease caused by the NoV strains most commonly associated with infection in humans. If future clinical trials confirm the efficacy of the NVLP vaccine in humans, large amounts of NVLPs will be needed to facilitate global vaccination campaigns.

The development of an effective NV vaccine has been hindered by the lack of an animal model for virus production and the inability to grow the whole virus in cell culture. Several expression systems have therefore been tested for the production of NVLPs, including baculovirus-infected insect cells, bacteria, yeast, mammalian cells, and plant-based systems. These production platforms have been investigated by electron microscopy to confirm the fact that the Norwalk virus coat protein (NVCP) self-assembles into NVLPs. Furthermore, the immunogenicity of the recombinant NVLPs has been investigated in animals.

The first attempt to produce NVCP in a heterologous system involved baculovirus-infected insect (*Spodoptera frugiperda*) cells (Sf9). NVLPs similar in size and appearance to native capsids were detected and, although no expression data were reported, the yield of purified protein ranged from 65 to 125 mg per liter of infected insect cell cultures [29].

NVCPs representing different NV strains have also been expressed in* E. coli* as fusion proteins with maltose binding protein (MBP) and thioredoxin. The yields of the purified fusion proteins were 26 and 56 mg/L, respectively, but no NVLPs were detected. The unassembled purified capsid proteins were analyzed to determine the possibility of establishing an immunologic detection system for NoV antigens, based on the enzyme-linked immunosorbent assay (ELISA), and confirming the diagnosis of NoV-infected patients using recombinant NVCP [49].

NVCP was successfully expressed in* P. pastoris* system after testing a range of expression vectors and culture conditions. Recombinant NVCP spontaneously formed NVLPs with final yield of 5–10 mg/L after purification. The yeast-derived NVLPs were tested as potential NV oral vaccines by feeding raw yeast extracts to animals. Even at doses as low as 0.1 mg, the yeast-derived NVLPs were able to induce significant systemic and intestinal mucosal responses in the animals [50].

Venezuelan equine encephalitis (VEE) virus replicon particles (VRPs) have been used as vectors to express NVCPs in BHK cells, resulting in the production of NVLPs with yields of approximately 10^10^ partially purified particles per mL [51]. VRPs can be used both as vectors to generate NVLPs in heterologous systems or as a self-replicating vaccine that produces recombinant NVLPs in target cells. Mice inoculated subcutaneously with these particles (two doses, 10^7^ infectious units each) developed systemic and mucosal immune responses to NVLPs, as well as heterotypic antibody responses to the major capsid protein from a different NV strain [52].

NVLPs have also been produced in many plant-based systems, with initial experiments focusing on constitutive expression in transgenic plants. Recombinant NVCP self-assembled into NVLPs that accounted for 0.23% of TSP in transgenic tobacco leaves [41] and NVCPs also accumulated to 0.37% of TSP in transgenic potato tubers (34 *μ*g/g of tuber weight) although only ~50% self-assembled into NVLPs. The oral immunogenicity of partially purified NVLPs from tobacco and potato was demonstrated in mice [41], whereas phase I clinical studies in humans demonstrated that the administration of uncooked potatoes containing NVLPs was safe, but only modestly immunogenic [45].

A modified NVCP gene, codon-optimized for plants, was later expressed in tomato and potato, resulting in the accumulation of NVCP at levels of up to 8% TSP in tomato fruits and 0.4% TSP in potato tubers, corresponding to 160 *μ*g/g (100 *μ*g NVLPs/g) in tomato fruits and 120 *μ*g/g (90 *μ*g NVLPs/g) in potato tubers. Freeze-dried potato and tomato tissues were immunogenic when fed to mice, but the delivery of the same doses of air-dried tomato fruit stimulated stronger immune responses. It was proposed that air-drying preserves the stability of NVLPs and the fruit tissue structure, thus conferring greater protection against proteolytic enzymes in the gut [53].

More recently, MagnICON vectors have been used for the rapid and efficient production of NVLPs in* Nicotiana benthamiana* plants. Different subcellular localizations were compared, and the highest yields were achieved by cytosol targeting (860 *μ*g NVCP/g FW in the leaves) at 12 days after infection (dpi). The partially purified recombinant NVLPs were orally immunogenic when fed to outbreed CD1 mice [54].

The agroinfiltration of* N. benthamiana* leaves with an optimized DNA replicon from bean yellow dwarf virus resulted in efficient replicon amplification and robust NVCP production within 5 days. The NVCP yield was ~340 *μ*g/g FW in the leaves and the protein assembled efficiently into NVLPs [55]. The same expression vector was recently used in lettuce, which produces low levels of secondary metabolites. This resulted in average NVCP yield of 200 *μ*g/g FW in the leaves [56]. The production of NVLPs in* N. benthamiana* plants has also been optimized using* Agrobacterium*-mediated transient gene expression for the simultaneous expression of two NV capsid proteins (VP1 and VP2) to increase NVLP stability, along with the* Pepper mild mottle virus* suppressor of viral posttranscriptional gene silencing. This achieved yields of up to 1 mg of partially purified NVLPs per g FW in the leaves [57].

The purification of NVLPs is usually achieved by using ultracentrifugation and density gradient methods that exploit particle size and density regardless of the expression platform [29, 41, 46, 51, 54, 55, 57]. However these methods are technically demanding and difficult to scale up, so alternative processing strategies have been explored [50, 56, 58]. Low-pH precipitation coupled with DEAE anion-exchange chromatography recently allowed the efficient purification of NVLPs from* N. benthamiana* leaves [59]. This was the first report to describe the scaled-up production of a pharmaceutical-grade (cGMP-compliant) NVCP vaccine in plants, and the product is currently being tested in a phase I human clinical trial.

## 4. Monoclonal Antibody 2G12 

The monoclonal antibody (mAb) 2G12 is a broadly neutralizing anti-HIV-1 human IgG1 that recognizes a high-mannose glycan cluster on the surface of the virus glycoprotein 120 (gp120). It was isolated from an asymptomatic HIV-1 infected patient in 1990, and in 1994 its neutralizing activity against HIV-1 strains and its ability to bind with gp120 were described for the first time [60, 61]. The broad biological activity of 2G12 allows it to defend against infection with primary HIV isolates from various clades, either by direct virus neutralization or by combination with other effector cells and complement activation [62].

As well as neutralizing HIV-1* in vitro*, passive transfer studies in primates demonstrated that 2G12 can control infection and prevent transmission* in vivo *following parenteral or mucosal administration, preferably in combination with other neutralizing antibodies [63, 64]. A phase I study in humans demonstrated the safety of repeated intravenous infusions of 2G12 combined with another broadly neutralizing antibody (2F5) when administered to asymptomatic patients infected with HIV-1 [65, 66]. Moreover, 2G12 combined with two broadly neutralizing antibodies (2F5 and 4E10) was able to delay viral rebound in patients whose infections were fully suppressed by antiretroviral treatment before antibody administration [67]. A phase II trial was then carried out to investigate the pharmacokinetic properties of the antibodies in the cocktail [68]. Such approaches require large doses of recombinant antibody (7–14 g of each antibody per patient) and thus create an immense demand, given that ~35 million people were living with HIV in 2012 (UNAIDS, 2013).

Broadly neutralizing human monoclonal antibodies such as 2G12 can also be applied as a mucosal microbicide to prevent HIV infection [69]. A recombinant form of 2G12 produced in stable transformed tobacco plants has been tested in a phase I clinical trial, based on intravaginal administration of the antibody to healthy female subjects at a dose range of 7–28 mg per individual (NCT01403792). Future trials will test the efficacy of prophylaxis in humans [70].

The complex and glycosylated structure of 2G12 means that it must be produced in eukaryotic expression platforms and then tested in specific assays to confirm its* in vitro *antigen-binding and neutralization capacity. The molecule was initially produced in hybridoma clones, generated by the electrofusion of B-cells and CB-F7 myeloma cells, producing 10 pg of the antibody per cell per day [60]. The mRNA for the 2G12 heavy and light chains was isolated and transcribed into cDNA in 1998 [71]. Large-scale antibody production was then achieved in CHO cells and the antibody was purified by protein A affinity chromatography. For most* in vivo* studies and clinical trials, 2G12 IgG1 was manufactured by Polymun Scientific Immunbiologische Forschung GmbH (Vienna, Austria) under cGMP guidelines, at a cost of €350–500/mg. Uniquely, the 2G12 prepared for clinical trial NCT01403792 was manufactured in tobacco leaves using a novel cGMP process.

HIV microbicides must be effective, safe, user-friendly, and above all economically affordable in the developing world, which has the highest number of HIV patients. Plants are ideal for the production of such low-margin/high-demand antibodies because of the economy of scale offered by agricultural production. This concept was developed in the EU project Pharma-Planta, which achieved the expression of 2G12 in several plant species and the fast-track development of transgenic tobacco as the primary production platform. The use of many different plants showed that the species, tissue, and subcellular compartment could affect the structure and composition of the antibody glycans, but this had no significant impact on the HIV-neutralization capacity of the antibody* in vitro* [72].

A secreted form of 2G12 has been constitutively expressed in the leaves of wild-type Arabidopsis plants [73] and in a mutant strain modified to knock out the genes encoding *β*1,2-xylosyltransferase (XT) and core *α*1,3-fucosyltransferase (FT), thus producing complex N-glycans lacking plant-specific residues [74]. The yield of the antibody was 0.05–0.2% of TSP in these young plants. The secreted and ER-retained versions of 2G12 were also produced in Arabidopsis seeds, achieving yields of 3.6 and 2.1 mg/g DW, respectively [75]. In the same series of experiments, the secreted form of 2G12 was also produced in the seeds of the XT/FT knockout line [75].

Cereals are considered more suitable for the production of recombinant proteins in developing countries because dry seeds preserve recombinant proteins in a stable form without a cold chain, and maize has been widely used for the production of pharmaceutical proteins in this context. A secreted form of 2G12 was expressed in the endosperm of the elite maize cultivar M37W and the best-performing line was passed through to a dedifferentiation-regeneration cycle, producing seeds yielding more than 100 *μ*g of the antibody per gram DW and eliminating most of the seed-to-seed variation [76]. HIV-neutralization assays showed that maize-derived 2G12 was nearly three times more potent than its CHO-derived counterpart, probably reflecting the higher proportion of aggregates (which are known to be more efficient than monomeric antibodies in terms of neutralization efficacy). The same antibody has also been retained in the ER of maize endosperm cells by adding a C-terminal KDEL tag to both antibody chains, resulting in its accumulation in ER-derived zein protein bodies [70]. These experiments were carried out using the cultivar Hill, but since this variety has little agronomic relevance, it was backcrossed to elite starch germplasms and a sugar-type sweetcorn background. The average yield in the T3 generation was 38.8 *μ*g/g DW, with a maximum of 60 *μ*g/g DW. As above, the plant-derived antibody was more potent in neutralization assays than the same antibody produced in CHO cells.

The 2G12 antibody has also been produced by transient expression in* N. benthamiana *leaves, initially using three glycoengineered lines in which RNA interference (RNAi) was used to suppress the synthesis of xylosylated and/or core *α*1,3-fucosylated glycan structures [77]. A binary vector carrying the cDNA sequences of both antibody chains was used for agroinfiltration and the yield was 110 *μ*g/g FW (corresponding to approximately 0.5% TSP) in the leaves. Similarly,* N. benthamiana *leaves were coinfiltrated with two binary vectors, one encoding the two antibody chains and the other carrying the p19 silencing suppressor gene [78]. Secreted and ER-retained forms of the antibody were produced with yields of ~100 *μ*g/g FW in the leaves at 6 dpi, increasing until 18 dpi. There was a small reduction in antigen-binding activity compared to 2G12 from CHO cells, probably reflecting the presence of residual impurities, but as above the HIV-neutralization capacity was higher. The 2G12 antibody has also been transiently expressed in* N. benthamiana *leaves using replicating and nonreplicating systems based on deleted versions of* Cowpea mosaic virus *(CPMV) RNA-2 [79]. In both cases, secreted and ER-retained versions of the antibody were expressed, yielding 14.8 and 37.8 *μ*g/g FW, respectively, using the replicating vector and 66.7 and 123.8 *μ*g/g FW, respectively, using the nonreplicating vector. The resulting antibody once again showed a marginally lower affinity for its antigen but similar or marginally better neutralization activity compared to 2G12 produced in CHO cells.

Tobacco has been used both for the transient and stable expression of 2G12. The secreted and ER-retained forms were transiently expressed in tobacco leaves coinfiltrated with* Agrobacterium tumefaciens *vectors containing expression constructs for the heavy and light chains, achieving yields of 80–100 *μ*g/g FW in the leaves [78]. The ER-retained form was expressed stably in transgenic tobacco leaves, accounting for 0.4% of TSP in the mature leaves [80]. As above, subsequent assays showed that the plant-derived antibody bound its antigen more weakly but neutralized HIV more potently than the CHO-derived counterpart. The expression of 2G12 in tobacco seeds achieved yields of 0.3% TSP, and immunolocalization studies demonstrated that the antibody accumulated in protein storage vacuoles (PSVs). The seed-derived antibody showed significantly lower antigen-binding activity than the leaf-derived protein, probably reflecting genetic segregation and thus the generation of a significant proportion of seeds expressing the heavy chain alone.

Finally, 2G12 has also been expressed in tobacco cell suspension cultures prepared from cultivar BY-2 [81]. Optimization of the nitrogen supply increased the yield to 12 mg/L by day 7 of the fermentation process. The antibody was secreted into the medium but a proportion also accumulated within the cells. The antigen-binding activity of the fully secreted antibody was 83% compared to the CHO counterpart (set arbitrarily at 100%), whereas that of the intracellular fraction was 40%. This probably reflects the fact that the intracellular antibody is a heterogeneous mixture containing all forms of the antibody at different stages of maturation, folding, and assembly, whereas only the fully folded and assembled version is secreted into the medium.

Several strategies have been proposed to improve the yield and stability of plant-derived recombinant proteins and reduce the costs of processing [2]. For example, the use of elastin-like polypeptides (ELPs) as fusion partners can increase the solubility and stability of recombinant proteins and facilitate purification by a process termed inverse transition cycling (ITC) [82]. Different versions of 2G12 have been expressed constitutively in transgenic tobacco leaves and seeds by fusing one or both antibody chains to ELPs, increasing the yields to 1% TSP [80]. Subsequent characterization of the purified antibodies demonstrated that the ELP fusion does not interfere with antibody assembly in tobacco and endows the recombinant antibody with greater antigen-binding activity albeit at the expense of HIV-neutralization efficacy. In the absence of a convenient fusion partner, the purification of antibodies such as 2G12 usually involves protein A affinity chromatography, an expensive processing option which achieves a recovery of 50–85% depending on the platform but is an expensive processing option. Therefore, additional nonprotein A protocols have been developed based on traditional chromatography methods, and these can achieve a recovery rate of 50–60% and a purity of up to 90% (e.g., [76, 83]).

## 5. Human Interleukin-6

Human interleukin-6 (hIL-6) is a 26-kDa secreted glycoprotein from the multifunctional cytokine family, which has diverse physiological roles including the induction of the acute phase response and inflammation, the regulation of the immune response, and the promotion of B-cell differentiation into immunoglobulin-secreting cells [84]. The hIL6 protein is also considered a myokine, that is, a cytokine produced by muscle cells in response to muscle contraction and physical exercise, stimulating lipolysis as well as fat oxidation [85, 86]. The overproduction of hIL-6 and other proinflammatory cytokines is associated with severe chronic immune-mediated inflammatory diseases (IMIDs) such as rheumatoid arthritis [87] and atherosclerosis [88]. The disruption of hIL-6 expression also occurs during the neurodegenerative process in Alzheimer's disease [89] and high levels of this molecule are associated with several hyperproliferative diseases and with the progression of cancer [90, 91].

The hIL-6 protein was first isolated from the supernatant of a T-cell line known as TCL-Na1, which is transformed with* Leukemia virus-1* [92], and its biochemical and functional characteristics were subsequently investigated [92, 93]. Native mature hIL-6 has two disulfide bonds and an N- glycosylation site at Asn73, although the glycan appears to be nonessential for biological activity. The activity of the protein can be evaluated* in vitro* by testing the stimulation of IgM production by SKW6-CL4 B-cells transformed with* Epstein-Barr virus* (EBV) [92] or the proliferation of mouse BALB/c lymphocytes or the hIL-6-dependent murine hybridoma cell lines B9 or MH60 [94].

Recombinant antibodies and synthetic peptides that target hIL6 and prevent interaction with its receptor (hIL-6R) are useful therapeutic candidates in rheumatoid arthritis, systemic-onset juvenile idiopathic arthritis (soJIA), and Castleman's disease [95, 96]. The development of such therapeutic molecules requires large quantities of functional hIL-6 but only small amounts of the native protein can be isolated from lymphocyte cultures, that is, ~3 *μ*g of pure protein from 5.7 L of culture medium [92]. The high cost of the recombinant protein produced in* E. coli* (~€10,000–15,000/mg) means that alternative platforms must be considered. Thus far, recombinant hIL-6 has been produced in* E. coli*,* P. pastoris*, baculovirus-infected insect cells, and tobacco plants.

The first attempts to express hIL-6 in* E. coli* involved the use of a pT9-11-derived plasmid with the inducible Trp promoter [97]. In attempting to express the mature protein with no signal peptide, trace amounts of hIL-6 were detected but no significant overexpression was observed. The first 20 amino acids of mature IL-2 (already overexpressed successfully in bacterial cells) were then added to the hIL-6 N-terminus along with a kallikrein cleavage site. The chimeric protein was expressed at high levels within inclusion bodies, with a final yield of 0.4 g/L. The mature form of hIL-6 was subsequently expressed as inclusion bodies using a synthetic gene with a codon-optimized N-terminal portion, with a final yield of 0.55 g/L and an estimated purity of ~60% [98, 99].

Two different approaches have been used to produce soluble hIL-6 in* E. coli*, one using an expression system designed to secrete the protein into the periplasmic space [100] and the other by fusing the protein to the secretion signal of bacterial *α*-hemolysin signal peptide for secretion into the culture medium [101]. In both cases, the protein was produced in a soluble and active form but with low yields (10 mg/L and 70 *μ*g/L, resp.). Soluble hIL-6 was subsequently expressed in* E. coli* strain BL21 as a fusion with MBP, thioredoxin, ubiquitin, or NusA, although only the MBP and NusA fusion constructs were successful. The maximum yield was 7.5 g/L in a bioreactor culture optimized for the NusA variant [102]. The authors did not report any attempt to remove the tag from the fusion protein and did not provide any information about the biological activity of the molecule.

More recently, a set of hIL-6 constructs with combinations of N and/or C terminal tags (His_6_, T7, GST, and the* E. coli* alkaline phosphatase periplasmic secretion signal) were expressed in different* E. coli* strains with reducing BL21 or oxidizing Origami 2 cytoplasmic environments, at different growth/induction temperatures, in the presence or absence of helper plasmids encoding cytoplasmic chaperones [103]. The highest yield of soluble hIL-6 was 2.6 mg/L and was achieved by expressing cytoplasmic hIL-6 in BL21 cells at 22°C in the presence of chaperones. The recombinant protein was active as shown by its ability to stimulate murine hybridoma cells.

Recombinant hIL-6 has also been expressed successfully in large-scale cultures of the methylotrophic yeast* P. pastoris* [104]. The mature protein cDNA was cloned in frame with the yeast *α*-factor secretion signal under the control of the inducible* AOX1* promoter (pPICZalphaA vector) and introduced into* P. pastoris* strain X33. Several culture and expression conditions were tested, both in shake flasks and in the large-scale bioreactor. The highest yield was achieved 96 h after induction, reaching 30 and 280 mg/L in the shake flasks and bioreactor, respectively. The molecular mass of the purified hIL-6 was ~20.9 kDa, indicating the absence of glycosylation. The bioactivity of hIL-6 produced in* P. pastoris* was five-fold higher than that of the commercial recombinant hIL-6 produced in* E. coli *when used to stimulate the growth of BALB/c mouse lymphocytes.

The first attempt to produce recombinant hIL-6 in baculovirus-infected insect cells involved the expression of full-length hIL-6 cDNA in a modified* Autographa californica nuclear polyhedrosis virus* (AcNPV) vector. This was used to transfect Sf9 cells, yielding modest amounts of a 22-kDa protein after 72 h, and partial purification of the protein was necessary to establish its biological activity [105]. Functional hIL-6 was subsequently expressed in baculovirus-infected Sf9 cells using a system based on inducible secretion, but the low yields (1 *μ*g/mL) were disappointing [106].

The production of a functional recombinant hIL-6 in transgenic tobacco plants was first reported by [107] but the yield was not determined. More recently, Nausch et al. [108] compared different transient and stable expression strategies for hIL-6 in tobacco and* N. benthamiana*. Stable expression was tested using three different constructs targeting the apoplast, ER, and vacuole, each controlled by the constitutive* Cauliflower mosaic virus* (CaMV) 35S promoter. The ER-retained version of hIL-6 accumulated to much higher levels (an order of magnitude higher) than the proteins targeted to the apoplast and vacuole, and the ER-retention construct was therefore selected. The three best-performing T0, expressing the ER-retained hIL-6, were self-pollinated to obtain T1 and T2 progeny, increasing the yield in the best-performing line to 1397 *μ*g/g TSP (112 *μ*g/g FW in leaves) and 1212 *μ*g/g TSP (303 *μ*g/g DW in seeds), respectively. The same construct was then used for transient expression in two tobacco cultivars and in* N. benthamiana* with the MagnICON system. Two different MagnICON systems were used, one based on the RNA-dependent RNA polymerase from* Turnip vein clearing virus* (TVCV) and the crucifer-infecting tobacco mosaic virus coat protein (cr-TMV/TVCV) and the other based entirely on* Potato virus X* (PVX). By using the cr-TMV/TVCV system, only cell-to-cell movement was observed in all the three host plants, whereas by using the PVX system the infection spreads systemically in* N. benthamiana* [109] but not in the two tobacco cultivars. The highest yields were achieved in* N. benthamiana*, where hIL-6 accumulated to 7.8% of TSP using the cr-TMV/TVCV system and 4.8% of TSP using the PVX system. Significantly lower values were achieved in tobacco, with a maximum yield of ~1% of TSP in the cultivar Virginia. The structural and biological properties of the recombinant proteins were tested by western blot analysis, revealing that plant-derived hIL-6 is present as two glycoforms (26-27 kDa) although no comparisons were made to the glycan structure on the native hIL-6 molecule. The activity of recombinant hIL-6 was tested by applying crude leaf extracts to mouse B-9 cells and performing a hybridoma proliferation assay, indicating that the activity of the plant-derived proteins was equivalent to the aglycosylated commercial standard hIL-6 produced in* E. coli* [94].

The processing strategy for hIL-6 is strongly dependent on the nature of the starting material. The insoluble hIL-6 recovered from* E. coli* inclusion bodies must undergo several solubilization/refolding steps based on the redox couple-assisted oxidation of cysteine residues, followed by a dilution or gel filtration refolding step. Additional ion exchange, reversed phase HPLC, and size exclusion chromatography steps may be used to increase product purity. Although highly pure (up to 99%) active hIL-6 can be recovered using these methods, the efficiency is often low, with a recovery rate of 15–20% [99]. The large number of purification steps and low final yield in these protocols is not cost-effective for industrial manufacturing, so a simpler protocol was developed for the isolation of soluble hIL-6 produced in* P. pastoris* [104]. Here the protein was purified from the culture supernatant by PEG precipitation, followed by anion-exchange and size exclusion chromatography, with a final yield of 56% and a purity of up to 95%. No purification strategy has yet been published for hIL-6 produced in plants.

## 6. Conclusions

In this review, we discuss the production of four recombinant proteins (hGAD65, NVLPs, 2G12, and hIL-6) which represent heterogeneous pharmaceutical applications, different biochemical features, and a corresponding wide range of production platforms.  Table 1 overviews the production of the target proteins in “traditional” heterologous expression systems. We have focused not only on the yields achieved in different production systems but also on the unique properties of the manufacturing process for each protein, thus highlighting the advantages of plant-based systems over fermenters for specific niche markets. This leads to the conclusion that plants are potentially most beneficial for the production of four major categories of pharmaceutical proteins:

1. pharmaceutical proteins required in large quantities, that is, commodity pharmaceutical proteins such as microbicide components;
2. pharmaceutical proteins that need to be produced rapidly, that is, rapid-response proteins such as vaccines against rapidly evolving viral strains;
3. biopharmaceuticals that require complex posttranslational modifications, that is, antibodies and recombinant proteins with specific glycan structures;
4. biopharmaceuticals intended for oral delivery.

Other plant-derived recombinant proteins, beyond the four targets described here, also fit in these categories, for example, vaccines against seasonal virus strains (e.g., full-length hemagglutinin protein from the A/Wyoming/03/03 (H3N2) strain of influenza, [110]), personalized vaccines (e.g., non-Hodgkin's lymphoma vaccines for individual patients, [111]), and proteins carrying specific glycans that increase their efficacy (e.g., human glucocerebrosidase for enzyme replacement therapy, [112]).

The large-scale production of recombinant pharmaceutical proteins is often hampered by the poor expression of their mature, active forms in prokaryotic hosts such as* E. coli* and by the high costs and the limited scalability of traditional fermenter-based platforms using mammalian cells. One of the most interesting issues that emerge from our case-by-case analysis is the high productivity of plants compared to other platforms when both the intrinsic yield (per unit biomass) and biomass yield (per hectare per year) are taken into account. This advantage is shown in Table 2 for the four case studies considered in this paper, in terms of the number of plants needed to produce 1 g of each target protein. The variety of available plant hosts and expression systems provide diverse toolbox for the manufacture of recombinant proteins. However, it is not a straightforward process to select the ideal plant-based expression system because many aspects need to be considered carefully, including product yields, quality, production scalability, costs, and cGMP compliance.

In our case studies, the highest yields were achieved in transgenic tobacco plants and by transient expression in* N. benthamiana*. For example, hGAD65 is expressed at higher levels in* N. benthamiana* than in tobacco (27.6 versus 10.5 *μ*g/g FW) but stably transformed tobacco plants are more productive overall because they produce much more biomass. Furthermore, the accumulation of hGAD65mut in tobacco leaves exceeds the levels achieved in* N. benthamiana* (143.6 versus 96.6 *μ*g/g FW), thus suggesting that the productivity would be higher in tobacco even without considering the enhanced biomass production [23]. Conversely, the accumulation of hIL-6 in* N. benthamiana* is up to 80-fold more than achieved by stable expression in tobacco leaves and seeds [102].

Meaningful comparisons among different platforms are required for proper evaluation but this is complicated by the diverse units used to report expression data. In our four case studies, yields were reported both in absolute units (mass of recombinant protein per unit of biomass, which allows total productivity to be calculated by factoring in the production scale) and in relative units (%TSP) which is less useful particularly when comparing dissimilar tissues such as leaves and seeds with vastly different water contents. If the costs of downstream processing are assumed to be the same for all platforms, the estimated costs for the manufacture of recombinant proteins in plants are much lower than current fermentation-based technologies because of the lower upstream costs [56]. For example, a 140-fold cost saving was estimated for the production of hGAD65 in transgenic tobacco compared to baculovirus-infected insect cells [23].

In addition to the flexible and cost-effective manufacturing offered by plants generally, transient expression systems offer the further advantage of rapid upscaling due to the short interval between transformation and expression. We used the* Norwalk virus* vaccine as a case study to highlight this niche. An effective vaccine needs to be produced quickly after strain identification in order to halt the spread of the new strain. NVLPs produced by transient expression address these issues, allowing the rapid and affordable production of strain-specific vaccines in a timely manner and in relevant locations, including the developing world [54, 55, 59, 110]. However, it should also be borne in mind that plants offer advantages for small-scale expression. For example, the production of personalized vaccines in mammalian cells would require a full production campaign and the “occupation” of a fermenter for each patient requiring a vaccine. In contrast, transient expression in plants would allow many similar vaccines to be prepared by using a small number of plants each enclosed in a protective chamber in a greenhouse. In this manner, the production of a customized idiotype vaccine for non-Hodgkin's lymphoma was achieved in less than two weeks from biopsy by using transient expression in plants [111, 113].

Another key advantage of plant-based expression platforms is their ability to synthesize complex eukaryotic glycan structures. Approximately one-third of approved biopharmaceuticals is thought to be glycoproteins, which favors the use of eukaryotic systems for such products [114]. Although N-glycan synthesis in the ER is conserved among eukaryotes, N-glycan processing in the Golgi body differs among phyla resulting in diverse glycan structures. Plant-derived glycoproteins typically contain non-human-glycans that are added in the Golgi body [114]. Whether these glycans are immunogenic or allergenic is still a matter of debate, but they can be immunoreactive [70].

Two of the target proteins considered in this review are glycosylated: 2G12 and hIL-6. The antibody 2G12 has been expressed in a wide range of plant-based platforms, including wild-type and glycoengineered systems, and has been targeted to different subcellular compartments, thus resulting in a huge variety of glycoforms. The detailed description of these data is beyond the scope of this paper, but overall it was found that the different glycan profiles do not affect the virus-neutralization activity of the antibody* in vitro *[72]. The impact of the different glycoforms* in vivo* should be considered in future studies, but it is likely that Fc-mediated antibody effector functions and antibody-dependent cell-mediated cytotoxicity could be influenced by different glycans and this should be considered in the context of systemic antibody administration [70]. Plant-derived hIL-6 was also produced as a number of different glycoforms, all of which had the same* in vitro *activity as the aglycosylated commercial counterpart produced in bacteria, but similarly the* in vivo* implications need to be evaluated. These considerations are less important if plant-derived pharmaceuticals are intended for topical application [117]. Importantly, plant-specific glycans may also be desirable in specific cases. For example, the presence of terminal mannose residues on plant-derived recombinant glucocerebrosidase was shown to increase its uptake by macrophages and thus its efficacy for the treatment of Gaucher's disease [112].

One final and unique advantage of plant-based systems is the natural “bioencapsulation” provided by edible plant organs when pharmaceuticals are intended for oral administration [45, 118]. The oral delivery of drugs is preferred [119] but unprotected peptides and proteins are exposed to the harsh gastrointestinal environment, which is acidic and rich in proteolytic enzymes. For oral vaccination, several phase I clinical trials have been carried out using edible plants, including those expressing NVLPs as discussed above [45]. These trials confirmed the safety and immunogenicity of NVLPs without the need for a buffer or vehicle other than the plant cell. Transgenic plants expressing antigens may therefore be a significant step towards the goal of developing cost-effective and user-friendly vaccines.

The oral route is also a potentially effective strategy for the prevention of autoimmune diseases by inducing tolerance. For example, the oral administration of plant tissue expressing hGAD65 (in combination with IL-4) to the nonobese diabetic mouse model of T1D effectively prevented the onset of the disease [21]. However, the clinical application of oral tolerance strategies in humans would be challenging because of the immense cost of autoantigen production, particularly if repeated regular doses are required to maintain the beneficial effects. Plants could meet this unprecedented demand for recombinant autoantigens, making such strategies safe, palatable, and economically feasible.

The examples discussed above can be considered proof-of-principle case studies that highlight some of the specific advantages of plant-based production platforms over traditional systems. It is unlikely that plants will completely replace CHO cells and other established systems, but they are now gaining a firm foothold in niche markets where the unique benefits of plants offer the greatest advantages.

## Figures and Tables

**Table 1:** Highest yields of the expression of the four selected recombinant pharmaceutical proteins in “traditional” heterologous production platforms.

Recombinant protein	Heterologous expression system	Highest expression level	Reference
hGAD65	*Escherichia coli *	12.5 mg/mL	[15]
	*Saccharomyces cerevisiae *	0.46 mg/mL	[27]
	*Pichia pastoris *	0.42 mg/mL	[27]
	*Spodoptera frugiperda* cells	0.02 mg/mL	[18]
	Mouse myeloma cells	1.67 mg/L	[16]

NVCP	Escherichia coli*	56 mg/L	[49]
	Pichia pastoris*	10 mg/L	[50]
	*Spodoptera frugiperda* cells*	125 mg/L	[29]
	Baby hamster kidney cells*	10^10^ particles/mL	[51]

2G12	Hybridoma clones	10 pg/cell/day	[60]

hIL-6	*Escherichia coli *	7.5 mg/mL	[102]
	*Pichia pastoris *	0.28 mg/mL	[104]
	*Spodoptera frugiperda* cells	0.001 mg/mL	[106]

*Reported values are the highest yield data of purified or partially purified recombinant protein because of the absence of expression data.

**Table 2:** Best-performing plant-based platforms for the production of four selected recombinant pharmaceutical proteins.

Plant host	Plant organ	Recombinant protein	Expression system	Highest expression level	Plants/g recombinant protein	Reference
Tobacco *(Nicotiana tabacum) *	Leaves	hGAD65mut	Transgenic	0.14 mg/g LFW	^ 1^93	[23]
		NVCP	Transgenic	0.2% TSP	ND	[41]
		IL6_KDEL	Transgenic	0.11 mg/g LFW	^ 1^119	[108]
		2G12/2G12_KDEL	Transient (binary vector)	0.1 mg/g LFW	^ 1^133	[78]
	Seeds	hGAD67/65mut	Transgenic	0.4 mg/g DSW	^ 1^1250	[26]
		IL6_KDEL	Transgenic	0.3 mg/g DSW	^ 1^1667	[108]
		2G12_KDEL/ELP	Transgenic	1.0% TSP	ND	[80]

*Nicotiana benthamiana *	Leaves	hGAD65mut	Transient (MagnICON vectors)	0.1 mg/g LFW	^ 2^3451	[23]
		NVCP (VP1 and VP2)	Transient (binary vector)	1 mg/g FLW	^ 2^333	[57]
		IL6_KDEL	Transient (MagnICON vectors)	7.8% TSP	ND	[108]
		2G12_KDEL	Transient (viral vector)	0.12 mg/g LFW	^ 2^2693	[79]

*Arabidopsis thaliana *	Seeds	hGAD67/65mut	Transgenic	4.5 mg/g DSW	^ 3^308	[26]
		2G12	Transgenic	3.6 mg/g DSW	^ 3^385	[75]
	Leaves	2G12	Transgenic	0.2% TSP	ND	[73]

Maize *(Zea mays*)	Seeds	2G12	Transgenic	>0.1 mg/g DSW	ND	[76]

Lettuce *(Lactuca sativa) *	Leaves	NVCP	Transient (viral vector)	0.2 mg/g LFW	ND	[56]

Petunia *(Petunia hybrida) *	Seeds	hGAD67/65mut	Transgenic	0.2 mg/g DSW	ND	[26]

Carrot *(Daucus carota) *	Taproots	hGAD65	Transgenic	0.01% TSP	ND	[22]

*Chlamydomonas reinhardtii *		hGAD65	Transgenic	0.3% TSP	ND	[20]

Potato plant *(Lycopersicon esculentum) *	Fruits	NVCP	Transgenic	0.16 mg/g fruit weight	^ 4^0.6	[53]

Tomato *(Solanum tuberosum) *	Tubers	NVCP	Transgenic	0.12 mg/g tuber weight	ND	[53]

The highest expression levels are reported as mass of recombinant protein per unit of biomass (LFW: leaves, fresh weight; DSW: dry seed weight) unless these values were not available, in which case percentage total soluble protein (%TSP) is used instead. The recombinant protein productivity values were calculated by considering the seed or leaf biomass yield per plant (^1^[115]; ^2^[59]; ^3^ [116];^ 4^ Mississippi State University website: http://msucares.com/crops/comhort/yield.html). ND: not determined (values were not calculated because of the absence of productivity data or the expression data were reported as %TSP).
